# Population level survival of patients with chronic myelocytic leukemia in Germany compared to the US in the early 21st century

**DOI:** 10.1186/1756-8722-6-70

**Published:** 2013-09-16

**Authors:** Dianne Pulte, Benjamin Barnes, Lina Jansen, Nora Eisemann, Katharina Emrich, Adam Gondos, Stefan Hentschel, Bernd Holleczek, Klaus Kraywinkel, Hermann Brenner

**Affiliations:** 1 Division of Clinical Epidemiology and Aging Research, German Cancer Research Center (DKFZ), Heidelberg, Germany; 2 Cardeza Foundation and Division of Hematology, Department of Medicine, Thomas Jefferson University, 1015 Chestnut Street Suite 1321, Philadelphia, PA 19107, USA; 3 National Center for Cancer Registry Data, Robert Koch Institute, Berlin, Germany; 4 Institute of Cancer Epidemiology, University of Lübeck, Lübeck, Germany; 5 Cancer Registry of Rhineland-Palatinate, Institute for Medical Biostatistics, Epidemiology and Informatics, University Medical Center, Johannes Gutenberg University Mainz, Mainz, Germany; 6 Hamburg Cancer Registry, Authority for Health and Consumer Protection, Hamburg, Germany; 7 Saarland Cancer Registry, Saarbrücken, Germany; 8 German Cancer Consortium (DKTK), Heidelberg, Germany

**Keywords:** Chronic myeloid leukemia, Survival, Period analysis, Prognosis

## Abstract

**Introduction:**

The advent of tyrosine kinase inhibitors has produced 5-year survival of 90 + % for chronic myelocytic leukemia (CML) patients in clinical trials. However, population level survival has been lower, especially in older patients. Here, we examine survival of patients with CML in Germany and compare it to survival of patients in the United States (US).

**Methods:**

Data were extracted from the Surveillance, Epidemiology, and End Results database in the US and 11 cancer registries in Germany. Patients 15–69 years old diagnosed with CML were included in the analysis. Period analysis for 2002–2006 was used to provide the most up-to-date possible estimates of five-year relative survival.

**Results:**

Five-year relative survival was 68.7% overall in Germany and 72.7% in the US. Survival was higher in the US for all age groups except for ages 15–39 years, but the difference was only statistically significant for ages 50–59 years (at 67.5% vs 77.7% in Germany and the US, respectively). Survival decreased with age, ranging from 83.1% and 81.9%, respectively, in Germany and the US for patients 15–39 years old to 54.2% and 54.5%, respectively, in patients 65–69 years old. Survival increased between 2002 and 2006 by 12.0% points in Germany and 17.1% points in the US.

**Conclusions:**

Five-year survival estimates were higher in the US than in Germany overall, but the difference was only significant for ages 50–59 years. Survival did not equal that seen in clinical trials for either country, but strong improvement in survival was seen between 2002 and 2006.

## Background

Treatment for chronic myelocytic leukemia (CML) has changed dramatically over the past several decades, especially in the early 21st century. Prior to the late 1980s, treatment was limited to chemotherapy, with a poor chance of 5-year survival for any patient subpopulation [1-3]. In the late 1980s-1990s, newer treatment options, including interferon-α [4,5] and hematopoietic stem cell transplant (HSCT) [6,7] became available, improving survival for some subpopulations. However, each of these treatment options is limited in its scope. Interferon-α is poorly tolerated in many patients, and only a subset of patients will respond. HSCT can be used only if a donor is available and, especially in the early years of HSCT development, only in younger and healthier patients. In 2001, the first targeted treatment for CML, imatinib, a tyrosine kinase inhibitor (TKI) [8], became available in Germany [9] and the US [10]. This medication was well tolerated and provided complete response in the majority of patients. Additionally, it can be used in sicker and older patients with less concern about toxicity. Clinical trials of CML patients in chronic phase treated with imatinib showed 5-year survival rates of nearly 90% and up to 95% when only CML related events were considered [11,12]. Newer tyrosine kinase inhibitors have shown even higher response rates and better survival, often with less toxicity [13,14].

Estimates of CML patient survival in the overall population have shown dramatic increases, especially for children and younger adults. However, survival has lagged in older adults. Additionally, the majority of population-level survival estimates for CML patients are derived from studies of the Surveillance, Epidemiology, and End Results (SEER) database in the United States [15-17] or from small series within Europe [18-20], with no prior studies concentrating specifically on CML patient survival in Germany. In the past, examination of population level survival with rare cancers in Germany has been hampered by lack of high quality data from a unified database. Recently, a collaborative project funded by the German Cancer Aid was set up between 11 population based German cancer registries and the German Cancer Research Center to provide comprehensive data on cancer survival in Germany [21]. This collaboration allows examination of detailed survival with rare cancers such as CML.

Here, we examine survival of patients diagnosed with CML in Germany between 1997 and 2006. Because age is an important prognostic factor in CML, we examine survival by age group as well as overall. Comparison is made to survival in the US using the SEER13 database.

## Methods

### Data sources

A detailed description of the cancer registries from which data were obtained has been published previously [21]. Briefly, data were extracted from 11 population-based cancer registries throughout Germany, representing a total base population of 33 million people (Table 1). Patients age 15 or older with a primary diagnosis of typical, BCR-ABL positive CML (ICD-10 code 92.1) between 1997 and 2006 and with vital status follow up through December, 2006 were included. For some registries, data were available starting from later years only. Because of data quality issues for patients age 70 years and older, in particular high proportions of cases notified by death certificate only (DCO), only data for patients 15–69 years old were included.

**Table 1:** Number of cases and percentage of cases diagnosed by death certificate only by registry of patients age 15–69 diagnosed with CML

**Registry**	**Underlying population in 2006 (millions)**	**Diagnosis period**	**Cases (1997–2006)**	**% DCO**^**a **^**(excluded)**	**Missing date (excluded)**	**Cases in the analysis**	**Median age at diagnosis**	**Microscopically confirmed cases**
Bavaria	8.13	2002-2006	285	15.4	0	241	55.0	100.0
Brandenburg	2.55	1997-2006	219	13.7	1	189	56.0	99.4
Bremen	0.66	1998-2006	53	15.1	0	45	62.0	95.6
Hamburg	1.75	1997-2006	160	5.6	0	151	53.0	100.0
Mecklenburg-Vorpommern	1.69	1997-2006	173	11.0	0	154	56.5	99.4
Lower Saxony	7.98	2001-2006	435	8.7	0	397	57.0	96.4
North Rhine-Westphalia	2.62	1997-2004	159	8.2	0	146	54.0	100.0
Rhineland-Palatinate	0.52	1998-2006	25	12.0	0	22	61.0	100.0
Saarland	1.04	1997-2006	94	1.1	0	93	58.0	100.0
Saxony	4.25	1997-2006	352	19.6	0	283	58.0	98.9
Schleswig-Holstein	1.85	1999-2006	157	19.1	0	127	58.0	100.0
Total	33.04		2112	12.5	1	1848	56.0	98.8

a = death certificate only.

In order to compare population-level survival for CML patients in Germany with survival in the United States (US), data from the Surveillance, Epidemiology, and End Results (SEER13) database were analyzed [22]. The same inclusion criteria as for patients from the German cancer registries were applied for the same time period. The SEER13 database includes data from 13 regional cancer centers in the US, covering a population of about 39 million people. Centers are chosen for inclusion based on their high quality and epidemiologically interesting population groups. The SEER population is considered to be similar to the general US population with respect to most sociodemographic characteristics, although it may be more affluent than average and may have slightly higher than average survival for some cancers [23].

### Statistical methods

Five-year relative survival estimates for the time period 2002–2006 were calculated using period analysis [24]. Period analysis, first introduced in 1996 [25], provides more up-to-date survival estimates than traditional cohort based analysis. In particular, it has been shown by extensive empirical evaluation, that period estimates of 5-year relative survival for a given period quite closely predict 5-year relative survival later observed for patients diagnosed during the period of interest [26,27]. In order to ensure comparability of survival estimates between both countries, age-adjusted survival estimates were derived by computing weighted sums of age-specific survival estimates using weights according to the proportion of cases in the age groups (15–39, 40–49, 50–59, 60–64, and 65–69) in Germany. Because survival of patients with CML varies with age and gender [28], we examined survival by major age groups as above and by gender. Differences in survival between men and women, as well as between patients in Germany and the US, were tested for statistical significance, overall and by single age groups, using model-based period analysis [29]. Additionally, model-based period analysis [29] was also employed to estimate changes in 5-year relative survival within the 2002–2006 period.

Relative survival was calculated as the ratio of actual survival to expected survival. Expected survival was estimated according to the Ederer II method [30] using national life tables. For German patients, life tables stratified by age, sex, and calendar year were obtained from the German Federal Statistical Office. Relative survival for the US patients was calculated using US sex-, age-, calendar year-, and race-specific life tables published by the Center for Disease Control (CDC) [31].

All calculations were carried out using SAS software (version 9.2), using macros developed for standard and modelled period analysis [27,32].

## Results

Overall, 2112 patients with CML diagnosed between the ages 15 and 69 years were identified in the German cooperative database, of whom 12.5% were reported by DCO, leaving 1848 for survival analysis (Table 1). During the same time period, 2919 cases, including 9 DCO cases (0.3%), were identified in the SEER13 database, leaving 2910 cases for analysis. The percentages of DCO cases by age for Germany and the US are described in Table 2.

**Table 2:** Percentages of cases diagnosed by death certificate only according to age in Germany and the US

	**Germany**	**US**
15-39	5.0	0.1
40-49	7.3	0.3
50-59	13.1	0.3
60-64	14.7	0.3
65-69	19.0	0.8
Overall	12.5	0.3

Overall five year relative survival was 68.7% in Germany and 72.7% in the US (Table 3). There was a trend towards higher survival for younger patients (15–39 years old) in Germany, although the difference did not reach statistical significance. For all other age groups, survival was higher in the US, although the difference reached statistical significance only for patients 50–59 years old for whom survival was 10.2 percentage points higher in the US (Table 3). Because there was some concern that the high rate of DCO cases among some registries may bias results, an analysis was performed excluding registries with more than 12% of cases being reported by DCO. The results did not materially change with the exclusion of high DCO registries, except for patients age 65–69, for whom the 5-year relative survival in Germany was lower than that calculated using all registries (Table 4).

**Table 3:** Five year relative survival of patients diagnosed with CML in Germany and in the US

		**Germany**				**US**		
**Age**	**N**	**RS**	**SE**	**N**	**RS**	**SE**	**Diff**	**P (Model)**
15-39	324	83.1	3.0	818	81.9	2.0	+1.2	0.9403
40-49	328	78.0	3.4	680	82.8	2.2	−4.8	0.1777
50-59	423	67.5	3.5	697	77.7	2.6	−10.2	0.0018
60-64	361	65.3	3.8	340	70.3	4.1	−5	0.2065
65-69	412	54.2	4.0	375	54.5	4.2	−0.3	0.5093
Overall	1848	68.7	1.6	2910	72.7^**a**^	1.5	−4	0.0036

a = age adjusted using the percentage of cases in each age group in Germany.

N = Number of cases.

RS = 5-year relative survival.

SE = Standard Error.

Diff = Difference between survival in Germany and the US.

**Table 4:** Five year relative survival of patients diagnosed with CML in Germany and in the US, excluding registries with more than 12% of cases reported by DCO

		**Germany**				**US**		
**Age**	**N**	**RS**	**SE**	**N**	**RS**	**SE**	**Diff**	**P (Model)**
15-39	175	81.0	4.6	818	81.9	2.0	−0.9	0.7043
40-49	176	77.0	4.8	680	82.8	2.2	−5.8	0.2409
50-59	218	68.6	5.0	697	77.7	2.6	−9.1	0.0310
60-64	174	61.0	5.5	340	70.3	4.1	−5.3	0.0623
65-69	220	45.3	5.7	375	54.5	4.2	−9.2	0.0009
Overall	963	65.6	2.3	2910	72.7^**a**^	1.5	−7.1	0.7043

a = age adjusted using the percentage of cases in each age group in Germany.

N = Number of cases.

RS = 5-year relative survival.

SE = Standard Error.

Diff = Difference between survival in Germany and the US.

Five year relative survival was higher for women than for men in all age groups in both Germany and the US, at 65.5% and 69.7% overall for men in Germany and the US, respectively, and 72.8% and 77% overall for women in Germany and the US, respectively (Table 5). For men, there was a trend towards higher survival in the US, with the exception of patients age 65–69, where both survival estimates were nearly equal. However, the finding of higher survival in the US was statistically significant (p < 0.05) only for the overall survival estimate (Table 5). For women, there was a trend towards higher survival in Germany for ages 15–39 years with a difference of +6.8 percentage points, but it did not reach statistical significance. For all other ages there was a trend towards longer survival in the US. The difference was most pronounced and statistically significant for age group 50–59 years at −14.6 percentage (Table 5).

**Table 5:** Five year relative survival of patients diagnosed with CML in Germany and the US for (a) men and (b) women, 2002–06

		**Germany**	**US**					
**Age**	**N**	**RS**	**SE**	**N**	**RS**	**SE**	**Diff**	**P (Model)**
a. Men								
15-39	207	78.4	4.1	528	80.6	2.6	−2.2	0.5275
40-49	184	76.5	4.5	383	80.5	3.1	−4	0.3275
50-59	234	65.1	5.1	401	73.9	3.5	−8.8	0.0575
60-64	207	61.5	5.6	191	68.8	5.5	−7.3	0.3367
65-69	242	50.3	5.5	204	48.9	5.7	+1.4	0.6480
Overall	1074	65.5	2.3	1707	69.7^**a**^	2.0	−4.2	0.0273
b. Women								
15-39	117	91.2	4.0	290	84.4	3.1	+6.8	0.2632
40-49	144	80.1	5.0	297	85.8	3.1	−5.7	0.3460
50-59	189	69.6	4.8	296	84.0	3.6	−14.6	0.0050
60-64	154	69.1	5.3	149	71.7	6.1	−2.6	0.4252
65-69	170	59.2	5.5	171	61.4	6.1	−2.2	0.6316
Overall	774	72.8	2.3	1203	77.0^**a**^	2.1	−4.2	0.0453

a = age adjusted using the percentage of cases in each age group in Germany.

N = Number of cases.

RS = 5-year relative survival.

SE = Standard Error.

Diff = Difference between survival in Germany and the US.

Survival improved between 2002 and 2006 in both Germany and the US (Table 6). In the US, the increase was statistically significant for both younger patients (15–59 years old) and middle-aged patients (60–69 years old), but was greater for middle-aged patients (Table 6). In Germany, the increase was statistically significant only for younger patients (+14.6 percentage points), although a trend towards increased survival was also observed for middle-aged patients (Table 6).

**Table 6:** Survival of patients diagnosed with CML in Germany and the US, 2002 versus 2006

	**2002**		**2006**			
**Age group**	**RS**	**SE**	**RS**	**SE**	**Diff**	**P (Model)**
Germany						
15-59	67.1	4.1	81.7	2.7	+14.6	0.0089
60-69	54.7	5.2	63.2	4.2	+8.5	0.2560
Overall	61.8	3.1	73.8	2.4	+12.0	0.0071
US						
15-59	74.3	2.6	86.5	1.9	+12.2	0.0010
60-69	49.4	5.2	73.7	4.4	+24.3	0.0027
Overall ^a^	63.2	2.6	80.3	2.1	+17.1	<0.0001

a = age adjusted using the percentage of cases in each age group in Germany.

RS = 5-year relative survival.

SE = Standard Error.

Diff = Difference between survival in Germany and the US.

## Discussion

Treatment for CML has changed substantially over the first few years of the 21^st^ century. Until 2001, the standard treatment for CML was hematopoietic stem cell transplant, if possible, and interferon-α with hydroxyurea and other chemotherapeutic agents used in cases of poor response or intolerance to interferon or lack of donor or inability to tolerate transplant. In May, 2001, imatinib, the first TKI specifically developed to block the activity of the Bcr-abl protein, was approved for use in CML in the United States [9]. It was approved for use in Germany in November, 2001 as well [8] and quickly gained acceptance as the new standard of care. The initial study of imatinib in chronic phase CML showed response rates of 90% or higher for recipients [11]. Subsequent studies of other TKIs have also shown very high response rates and durable remissions [12,13]. However, population level data have generally shown a discrepancy between the results obtained in these trials and survival in the general population, especially in older patients [3,15,16]. Although use of imatinib in older patients has been shown to be safe and effective in clinical trials [33,34], its use in older patients in the general population has lagged [17].

The improvement in survival between 2002 and 2006 may be partly driven by increased uptake of imatinib in that time period and possibly to initiation of the medication sooner after diagnosis, specifically use on patients still in the chronic phase where it is most effective. Although there is little data directly addressing uptake of specific medications in different countries, one study suggests that there was rapid uptake of imatinib in both Germany and the US [35]. Additionally, a survey of physicians in the US and Europe (including, but not limited to, Germany) regarding practice in 2005–06 showed that physicians surveyed in each location chose TKI for first line therapy in most situations, including treatment of older patients. Overall, major differences in practice were not observed. However, use of pre-TKI options after failure of first line TKI in older patients (i.e. interferon or hydroxyurea) was more common among European physicians, possibly suggesting a lower uptake of second generation TKI, especially for older patients [36]. It should be noted that more physicians in Europe were familiar with testing for mutations in Bcr-abl that lead to TKI resistance, possibly explaining the differences in therapy plans observed. Since this study did not specifically examine treatment patterns in Germany, it is not possible to say whether the results are reflective of standard practice in Germany versus other parts of Europe. Additionally, physicians who responded to the survey may not represent typical practice as they are to some degree self-selected.

Because period analysis of survival in 2002–2006 uses data from patients diagnosed as early as 1997, the extra six months of generally available imatinib in 2001 may also give the US an advantage in terms of survival estimates, especially in, but not limited to, earlier time periods. Additional factors, including later diagnosis, differences in tumor biology, and random fluctuation of response in a rare disease should also be considered. A larger percentage of German patients were diagnosed at age 60–69, which may suggest delay in diagnosis in Germany or possibly differences in tumor biology. However, it should be noted that the median age of the population in Germany is older than in the US and thus it is difficult to draw definitive conclusions from this finding.

Strengths of our study include the use of large databases with survival information from a broad sampling of each country, allowing for evaluation of survival and subgroup analysis of this rare tumor and decreasing the risk that survival differences between individual parts of the country will unduly influence the impression of survival in the country overall. Use of population-level data provides a more realistic picture of diffusion of changes in the standard of care and of how probable a patient with a given disease in the general population is to survive in contrast to data provided by clinical trials which tend to present the best-case scenario with respect to survival and may not be realistic for the general population. Additionally, the use of period analysis and modelled period analysis provides the most up-to-date estimates of survival possible.

In interpreting our results, several limitations should also be considered. First, despite the large population bases, the relative rarity of CML limits our ability to detect minor differences in survival. Second, in the absence of a national death index in Germany, most cancer registries rely on record linkage with vital statistics from the (federal) state that they cover and may miss deaths among patients who move out of the state. Nevertheless, previous validation studies have suggested potential overestimation of survival due to deaths missed by migration to be very small [21]. Third, data on chemotherapy administration or oral therapeutic medications is available in neither the SEER nor the German database and therefore we can not make definitive statements about how use of specific medications affects survival. Fourth, there is some evidence that survival estimates from the SEER database may be higher than survival in the US population in general [23], so some caution is necessary when comparing survival in the two countries. Fifth, because some of the German databases are fairly new, the analysis is restricted to 5-year survival which prohibited analysis of longer survival, such as 10-year relative survival, that is of particular interest in CML. Additionally, data in this project were available only through 2006 so newer developments since the introduction of second-generation TKIs are not yet captured.

Finally and most significantly, several of the newer German registries are still in the build-up phase and the proportion of cases identified by DCO is still rather high in some of these registries. Exclusion of these DCO cases in the analysis may lead to some overestimation of survival [37,38]. Exploring the potential magnitude of such overestimation by plausibility ranges [39] suggests that true survival in Germany might be up to a few percentage points lower than estimated. Thus, the survival gap between Germany and the US might be even somewhat larger than suggested by the available data, especially in the older age groups in which DCO proportions are highest. This suggestion was supported by sensitivity analyses excluding cancer registries with DCO proportions >12% which yielded somewhat lower survival estimates in Germany, especially in age group 60–69 years. Additionally, the large proportion of DCO cases in older patients (age 70+) from Germany precluded sufficiently reliable analyses for this age group which means that no conclusions can be drawn with respect to this important segment of the population.

In summary, 5-year relative survival for patients with CML is high, especially for younger patients, in both the US and Germany, although survival is higher in the US for some age groups. Survival is improving in both countries, with greater improvement seen among middle-aged patients (age 60–69) in the US, perhaps suggesting improved uptake of TKIs among these patients. Nevertheless, prognosis in this age group continues to lag behind prognosis in younger patients. Further emphasis on treatment of middle aged patients with CML and potentially further developments in the treatment of CML may continue to improve survival in this age group.

## Competing interests

The authors have no conflicts of interest to disclose.

## Authors’ contributions

DP, BB, and HB designed the research. LJ performed the research. All authors contributed to the analysis of the data and review of the manuscript. DP wrote the manuscript. All authors read and approved the final manuscript.
